# A mixed population of *Helicobacter pylori, Helicobacter bizzozeronii* and “*Helicobacter heilmannii*” in the gastric mucosa of a domestic cat

**DOI:** 10.1186/2046-0481-67-25

**Published:** 2014-11-19

**Authors:** Rute Canejo-Teixeira, Manuela Oliveira, Hugo Pissarra, Maria Manuela Manuela E E Niza, Christina L Vilela

**Affiliations:** Faculdade de Medicina Veterinária, Universidade de Lisboa, CIISA, Avenida da Universidade Técnica, Lisboa, 1300-477 Portugal

**Keywords:** Feline, *Helicobacter*, Zoonosis, Gastritis, PCR, Histopathology

## Abstract

**Background:**

The presence of *Helicobacter* within the gastric mucosa is responsible for producing pathology in many animal species, including man. Since humans have been shown to harbour many of the same bacterial species as domestic carnivores, concern over their zoonotic potential has been growing. *Helicobacter pylori*, a class 1 carcinogen responsible for cases of gastritis and gastric cancer in humans, produces similar pathology in pet carnivores and is considered an example of anthroponosis. The case here presented refers to a 13 year-old mixed breed spayed female cat seen at necropsy.

**Findings:**

Stomach samples were analysed for the presence of *Helicobacter* spp. by cytology, histopathology and PCR. Mild mucosal atrophy was observed in the fundus and antrum, while lymphoplasmocytic infiltrates where noted in the lamina propria of the antrum. *Helicobacter*-like organisms were observed in the corpus and antrum, occupying gastric glands and surface mucosa. It was possible to detect *Helicobacter* spp., *H. pylori*, *H. heilmannii* and *H. bizzozeronii* in the fundus, corpus and antrum by PCR, while in the antrum PCR samples were positive for *H. pylori*.

**Conclusions:**

The spayed female under study could represent either a yet un-described population of domestic cats infected with *H. pylori* or a case of anthroponosis.

## Findings

### Background

*Helicobacter pylori* is a gram negative, urease positive, spiral bacteria classified by the World Health Organization as a class 1 carcinogen as its relationship to human gastritis and gastric cancer has been firmly established [1]. The majority of the 32 species described to date are enteric microorganisms of mammals, while 12 species are gastric inhabitants. Four species are now considered to be common in the gastric mucosa of domestic carnivores, *Helicobacter felis*, *Helicobacter bizzozeronii*, *Helicobacter salomonis*, *“Helicobacter heilmannii”* types 1, 2 and 4 [2, 3], while the role of *H. bilis’* as a primary gastric organism remains questionable. Although the pathogenic role of these species in gastritis and/or gastric cancer has yet to be firmly established in pets [4, 5], felines seem to be much more susceptible then canines [6]. Gastric fibroses and atrophy have been linked to *H. felis* infection in cats [7] while the presence of lymphoid follicles and inflammation have been related to *Helicobacter* spp. and *“H. heilmannii” senso lato* colonization [8, 9].

It has been suggested that, similarly to *H. pylori* infected humans, the presence of *H. heilmannii* could play a role in feline alimentary lymphoma [10]. There are several reasons for the growing concern regarding the zoonotic potential of these bacteria [11]. Firstly, humans have been shown to harbour species other than *H. pylori* with pathological consequences; secondly, no environmental source for these bacteria has been found; and lastly, epidemiological studies have shown a link between animal contact and infection [12]. Although *H. pylori* produces similar pathology in pet carnivores and in humans, this species has yet to be found in normal domestic carnivore populations [13, 14], making these cases good examples of anthroponosis [4, 6, 13]. However, Buczolits et al. [15] have identified two sequences from *Helicobacter*-like organisms 100% identical to *H. pylori* in the gastric mucosa of canines, re-kindling the debate on the role of pet carnivores in the transmission of this bacterium to humans. The present work describes the presence of *H. pylori* in a mixed population of *Helicobacter* species in the gastric mucosa of a domestic short-hair cat.

### Case presentation

A 13 year-old mixed breed spayed female cat with a history of mammary tumors and pleural effusion, euthanized at the owners’ request, was presented for necropsy to the Pathology Department of the Faculty of Veterinary Medicine, University of Lisbon. The stomach was opened along the greater curvature, brush cytology was performed and four full thickness biopsies where obtained from the fundus, corpus and antrum for histopathology and PCR analyses. Brush cytologies where obtained using sterile, single use inoculation loops and stained with May-Grünwald-Giemsa. One biopsy from each gastric region was stored in 10% formalin for histology processing. A combined sample, comprising one sample from each region, and the remaining biopsies from the three regions were kept frozen at −80°C, until DNA extraction with Qiagen DNeasy® Blood & Tissue Kit, according to the manufacturer’s instructions. PCR reactions for *Helicobacter* spp*.*, *H. pylori*, *H. felis*, *H. heilmannii*, *H. bizzozeronii*, *H. salomonis* and *H. bilis* were performed as previously described [3, 7, 16–19], using FidelTaq™ MasterMix (USB® Products – Affymetrix, Inc.). Reference strains *H. pylori* CCUG 17874 T, *H. felis* ATCC 49179, *H. bizzozeronii* CCUG 35045, *H. salomonis* CCUG 37845, *H. bilis* ATCC 51630 and *H. heilmannii* DNA (kindly provided by Professor K.W. Simpson) were used as positive controls. Biopsies for histopathology were imbedded in paraffin blocks, processed and stained with Hematoxylin and Eosin and with Giemsa.

**Figure 1:**
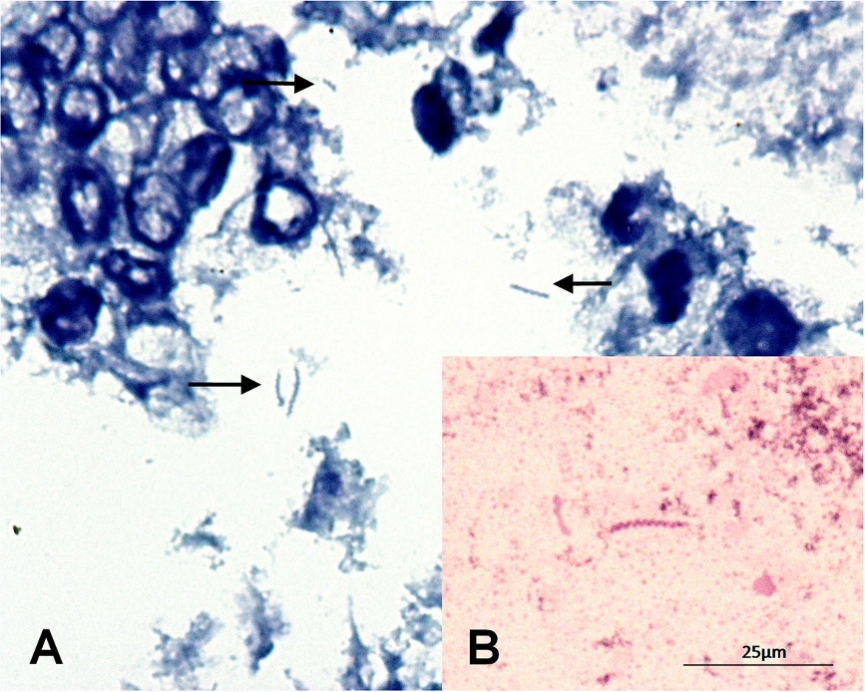
**Spiral bacteria, consistent with Helicobacter spp. found in the stomach (A) in the surface mucosa.** 1000x. Giemsa. **(B)** and brush cytology of the fundus region. 1000x. May-Grünwald-Giemsa. (Original photographs).

**Figure 2:**
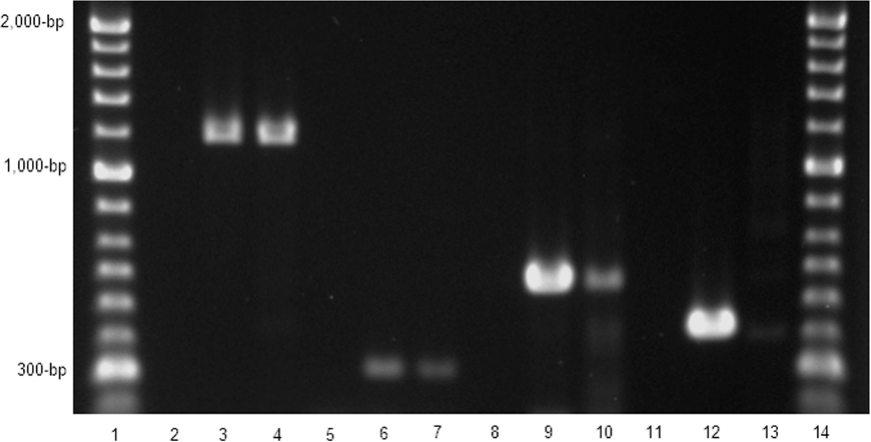
**PCR reactions.** Molecular ladder in lane 1 and 14 (Hyperladder II ™ Bioline); negative controls in lane 2, 5, 8 and 11; positive controls *Helicobacter* spp. (lane 3 using *H. felis* ATCC 49179), *H. pylori* (lane 6), *H. heilmannii* type 2 (lane 9), *H. bizzozeronii* (420-bp, lane 12); sample results *H.* spp (1,200-bp, lane 4), *H. pylori* (298-bp, lane 7), *H. heilmannii* (580-bp, lane 10) and *H. bizzozeronii* (420-bp, lane 13).

Based on the World Small Animal Veterinary Association histopathological standards [20], mild mucosal atrophy was observed in the fundus and antrum, while moderate lymphoplasmocytic infiltrates were noted in the lamina propria of the antrum. *Helicobacter*-like organisms were observed in the corpus and antrum, occupying gastric glands and surface mucosa (Figure 1A), where colonization was classified as mild. Brush cytology was positive in the fundus and corpus (Figure 1B), but negative in the antrum. PCR performed using DNA extracted from the combined sample was positive for *Helicobacter* spp., *H. pylori*, *H. heilmannii* and *H. bizzozeronii*, originating 1200-bp, 298-bp, 580-bp and 420-bp amplicons, respectively (Figure 2). When tested individually, the three regions were positive for *Helicobacter* spp*.*, *H. heilmannii* and *H. bizzozeronii*, while only the antrum was positive for *H. pylori*.

## Conclusions

The presence of a *Helicobacter* mixed population has been previously described in feline gastric biopsies [21]. Our finding of mild mucosal atrophy is consistent with other studies [6, 22]; however Simpson et al. [7] linked changes in the architecture of the gastric mucosa with the presence of *H. felis*, a species not found in this cat. *H. heilmannii* has been shown to alter gastric architecture but through epithelial proliferation and lymphoid follicular hyperplasia [9], not atrophy. It is possible however that the presence of *H. pylori* and *H. bizzozeronii* altered the pathogenic capacity of *H. heilmannii* as described for mixed infections of *H. bizzozeroniiI/H. felis* [11]. The fact that the fundus and antrum were the most affected regions is consistent with others findings [8] and, although not characteristic, the presence of inflammation in the antrum is similar to that found in felines experimentally infected with *H. pylori* [21].

The occurrence of *H. pylori* in the gastric mucosa of felines has been only reported in a particular commercial breeder [23] but not in stray and domestic populations [13, 14, 24]. The spayed female under study could represent either a yet un-described population of domestic cats infected with *H. pylori* or a case of anthroponosis, as hypothesized by some authors [4, 5, 13]. Considering *H. pylori*’s ability to survive in water [25] and the tendency for keeping indoor/outdoor cats in Portugal, colonization of this animal through a contaminated water source must also be considered. It was not possible to determine whether human co-inhabitants were *H. pylori* positive or if the animal had outdoor access.

The presence of *H. heilmannii* in cats has been linked to various alterations in gastric mucosa [9] and alimentary lymphoma [10]. Jergens et al. [22] have shown that treatment leads to the improvement of gastritis clinical signs and bacterial clearance, although histological signs of gastritis remain, suggesting a causal relationship similar to that seen in *H. pylori* infected humans [16]. However, as other studies could not evidence such relationship, other factors such as the effect of mixed infections and the possibility of strain dependent virulence, should be considered. The presence of a mixed infection of *H. heilmannii* and *H. pylori*, as found in this cat, could explain the development of pathology in some animals and not in others infected only with *H. heilmannii*. Given *H. pylori’s* focal distribution pattern [6], the small sample size normally obtained through endoscopy and even biopsy [21] may explain the high rate of negative results for *H. pylori*.

The role played by *Helicobacter* in feline gastritis, associated or not with lymphoma, remains controversial. Several factors, such as the species of *Helicobacter* present, the virulence of the strain, and the genetic predisposition of the animal, should be further investigated in order to better understand their relation to clinical disease.

## Abbreviations

PCR: Polymerase chain reaction
DNA: Deoxyribonucleic acid
Bp: Base-pairs.
